# Descriptive Epidemiology of Fabry Disease Among Beneficiaries of the Specified Disease Treatment Research Program in Japan

**DOI:** 10.2188/jea.JE20110101

**Published:** 2012-07-05

**Authors:** Kazuya Tsuboi, Sadao Suzuki, Masaki Nagai

**Affiliations:** 1 Department of Hematology, Nagoya Central Hospital, Nagoya, Japan; 2 Department of Public Health, Graduate School of Medical Sciences, Nagoya City University, Nagoya, Japan; 3 Department of Public Health, Saitama Medical University, Iruma-gun, Saitama, Japan

**Keywords:** Fabry disease, Japan, epidemiology, Specified Disease Treatment Research Program

## Abstract

**Background:**

Fabry disease (FD) is a rare X-linked lysosomal storage disorder and is included in the Specified Disease Treatment Research Program in Japan, which subsidizes medical care for beneficiaries with rare and other, designated diseases. However, no report on the epidemiologic features of Fabry disease has been published in Japan.

**Methods:**

We used clinical research data reports submitted to the program between 2003 and 2008 to assess the epidemiologic features of 315 beneficiaries with FD.

**Results:**

Of the 315 program beneficiaries, 198 were men (mean age, 37.4 years) and 117 were women (mean age, 51.2 years). The overall incidence in Japan was 0.25 cases per 100 000 individuals, and prevalence among men was 1.78 times that among women. More than 80% of beneficiaries were capable of working, going to school, or doing housework; however, 46 beneficiaries (14.6%) required home care, and 9 (2.9%) were living in hospitals or other medical facilities. As compared with the previous year, the clinical course of FD at beneficiary registration was unchanged for 178 of 290 beneficiaries (61.4%), worse for 81 (27.9%), and improved or cured for 31 (10.7%). The distribution of beneficiary-related characteristics was similar between men and women, and no significant difference was observed.

**Conclusions:**

The high percentage (>80%) of individuals with FD who were able to work, attend school, and perform tasks such as housework could reflect an improvement in the clinical course of FD after enzyme replacement therapy. We must continue data collection and conduct further studies to improve our understanding of the descriptive epidemiology of FD.

## INTRODUCTION

Fabry disease (FD) is a rare X-linked lysosomal storage disorder caused by a deficiency of the enzyme α-galactosidase A.^1^ FD causes glycolipids such as globotriaosylceramide to accumulate in the vascular endothelium of several organs, including the skin, kidneys, nervous system, and heart, thereby triggering inflammation and fibrosis.^2^ These processes generally result in organ dysfunction, which is usually the first clinical evidence of FD. Patients with classic FD have various symptoms, eg, acroparesthesias, hypohidrosis, angiokeratomas, corneal opacities, cerebrovascular lesions, cardiac disorders, and renal dysfunction.^1^ The symptoms are progressively debilitating, reduce the ability of patients to attend work or school, and severely affect quality of life (QOL).^3^

The prevalence of FD is estimated to be between 0.85 and 2.5 cases per 100 000 individuals worldwide.^4^ Onset of symptoms generally occurs during childhood, and, by middle age, life-threatening complications often develop in untreated patients.^2^ Life expectancy is reduced by approximately 20 years in untreated men and by about 15 years in untreated women.^5^^,^^6^ Treatment of FD includes enzyme replacement therapy with agalsidase alpha or beta, which have been used in more than 2000 patients to date.^7^ Therapy must be initiated early to prevent irreversible organ damage and continued deterioration of QOL. Because of the low prevalence of FD, identification of patients is a challenge and is further hindered by the lack of epidemiologic studies. Studies have been limited to small populations of high-risk patients with comorbidities, including those undergoing hemodialysis^8^^–^^10^ and those with a history of ischemic stroke,^11^ although programs to screen newborns may be an effective strategy in the future.^3^

In Japan, FD is included in the Specified Disease Treatment Research Program, which subsidizes medical care for beneficiaries with rare and other, designated diseases. As part of the program, the national government and prefectures assume the patients’ share of medical expenses for 56 specified diseases—the so-called intractable diseases (*nanbyo* in Japanese). The system is explained in detail elsewhere.^12^ In brief, specified illnesses include diseases that develop from an unidentifiable cause and are difficult to treat without an established treatment procedure. The diseases are chronic and may have serious consequences that make it difficult or impossible for patients to return to work or school. To benefit from the program, after a doctor diagnosis, a patient must submit an application to the government of the prefecture in which he or she lives. Clinical information is then entered into a database, and the data are collected by the Health Service Bureau of the Ministry of Health, Labour and Welfare. If the application is accepted, a Certificate indicating that the person is a Recipient of Designated Disease Treatment is issued.

To our knowledge, no epidemiologic report on FD in Japan has been published. The objective of this study was thus to investigate the basic characteristics and epidemiologic features of FD among beneficiaries, using the collected clinical data.

## METHODS

### Subjects

Analysis was performed using data from 315 individuals with FD who were beneficiaries of the Specified Disease Treatment Research Program. Data were obtained from clinical research reports submitted by beneficiaries who applied to the program between 2003 and 2008.

### Epidemiologic features

The primary measures included basic demographic characteristics, including sex and age distribution, and epidemiologic features such as age at onset, number of beneficiaries by geographic region, daily life status, status of care-requirement certification, status of certification of physical disability, and clinical course as compared with the previous year. A standardized prevalence ratio was calculated by using an indirect method for age standardization in 9 Japanese regions: Hokkaido, Tohoku, Kanto, Koshinetsu/Hokuriku, Tokai, Kinki, Chugoku, Shikoku, and Kyushu. Population data were collected from the 2005 census. Differences between men and women were evaluated using the *t* test or chi-square test. All *P* values were 2-sided, and a *P* value less than 0.05 was considered to indicate statistical significance. All analyses were performed with SAS version 9.1 (SAS Institute, Inc., Cary, NC, USA).

### Ethical considerations

This study was conducted with the approval of the ethics committees of the Nagoya City University and Nagoya Central Hospital according to the Declaration of Helsinki, the Ethical Guidelines for Clinical Studies from the Japanese Ministry of Health, Labour and Welfare, and the Ethical Guidelines for Epidemiological Research from the Japanese Ministry of Education, Culture, Sports, Science and Technology and the Ministry of Health, Labour and Welfare. Names and addresses were removed from the files when data were entered into the database. Therefore, information that could identify individuals was not included in the analyzed data. All subjects provided written consent for their data to be included in the study.

## RESULTS

Data were available for 315 men and women with FD who were beneficiaries of the Specified Disease Treatment Research Program between 2003 and 2008. The population sample included 198 men and 117 women, and there were 30 to 50 new beneficiaries per year. Table 1 shows the age distribution of beneficiaries at registration and at disease onset by sex. Men were generally aged 20 to 40 years at registration, whereas women tended to be in their 50s. Average age at registration was significantly lower for men (mean ± SD, 37.4 ± 13.9 years) than for women (51.2 ± 14.1 years, *P* < 0.0001). Average age at disease onset was also lower in men (19.6 ± 16.4 years) than in women (30.7 ± 19.6 years, *P* < 0.0001).

**Table 1.** Age distribution of male and female beneficiaries at registration and disease onset

Age (years)	Age distribution at registration						Age distribution at disease onset					
	
	Men		Women		Total		Men		Women		Total	
					
	Number	(%)	Number	(%)	Number	(%)	Number	(%)	Number	(%)	Number	(%)
<10	3	(1.5%)	0	(0.0%)	3	(1.0%)	35	(26.5%)	7	(9.3%)	42	(20.3%)
10–19	15	(7.6%)	6	(5.1%)	21	(6.7%)	44	(33.3%)	17	(22.7%)	61	(29.5%)
20–29	50	(25.3%)	4	(3.4%)	54	(17.1%)	17	(12.9%)	8	(10.7%)	25	(12.1%)
30–39	50	(25.3%)	13	(11.1%)	63	(20.0%)	16	(12.1%)	9	(12.0%)	25	(12.1%)
40–49	39	(19.7%)	18	(15.4%)	57	(18.1%)	8	(6.1%)	20	(26.7%)	28	(13.5%)
50–59	26	(13.1%)	45	(38.5%)	71	(22.5%)	5	(3.8%)	8	(10.7%)	13	(6.3%)
60–69	14	(7.1%)	22	(18.8%)	36	(11.4%)	6	(4.5%)	3	(4.0%)	9	(4.3%)
≥70	1	(0.5%)	9	(7.7%)	10	(3.2%)	1	(0.8%)	3	(4.0%)	4	(1.9%)

Total	198	(100%)	117	(100%)	315	(100%)	132	(100%)	75	(100%)	207	(100%)

Although most male beneficiaries (59.8%) developed FD before age 20 years, the age at which female beneficiaries were diagnosed was more variable, and 45.3% of women first displayed symptoms at age 40 years or older. Duration of FD was also assessed among beneficiaries. Although disease duration was usually less than 30 years among male beneficiaries (87.3% for <30 years, 11.9% for 30–49 years, and 0.8% for ≥50 years), it was more variable among female beneficiaries (78.4% for <30 years, 16.3% for 30–49 years, and 5.4% for ≥50 years).

Table 2 shows FD prevalence by region. Overall prevalence in Japan was 0.25 cases per 100 000 individuals (2005 census data), and FD prevalence among men was 1.78 times that among women. By region, Tohoku had the highest prevalence (0.37 per 100 000), followed by Koshinetsu/Hokuriku (0.35 per 100 000) and Tokai (0.31 per 100 000); FD was extremely uncommon in Hokkaido, which had only 2 cases (0.04 per 100 000). The standardized prevalence ratio was also highest in Tohoku (1.57, *P* < 0.05), followed by Kinki (0.59, *P* < 0.05), and lowest in Hokkaido (0.15, *P* < 0.05). Excepting these 3 regions, the standardized prevalence ratio was not statistically significant. The distribution by region was generally similar for male and female beneficiaries.

**Table 2.** Prevalence of Fabry disease by Japanese region, based on the number of beneficiaries identified from clinical research data as part of the Specified Disease Treatment Research Program and Japanese population data (2005 census)

	Males			Females			Total		
			
Region of Japan	Number	Prevalence^a^	Standardized prevalence ratio	Number	Prevalence^a^	Standardized prevalence ratio	Number	Prevalence^a^	Standardized prevalence ratio
Hokkaido	1	0.04	0.12^b^	1	0.03	0.18^b^	2	0.04	0.15^b^
Tohoku	23	0.50	1.64^b^	13	0.26	1.45	36	0.37	1.57^b^
Kanto	69	0.33	1.00	43	0.21	1.15	112	0.27	1.06
Koshinetsu/ Hokuriku	20	0.48	1.58	10	0.23	1.28	30	0.35	1.47
Tokai	27	0.36	1.14	20	0.26	1.49	47	0.31	1.27
Kinki	21	0.21	0.66^b^	9	0.08	0.47^b^	30	0.14	0.59^b^
Chugoku	9	0.24	0.81	3	0.08	0.43^b^	12	0.16	0.66
Shikoku	6	0.31	1.03	6	0.28	1.56	12	0.29	1.24
Kyushu	22	0.32	1.02	12	0.15	0.87	34	0.23	0.95

Total	198	0.32		117	0.18		315	0.25	

^a^Per 100 000 individuals in 2005.

^b^Significantly higher or lower (*P* < 0.05).

Beneficiary-related characteristics such as activities of daily life, medical services, physical-disability certification, certified-care requirements, and clinical course are shown in Table 3. The effect of FD on daily life was evaluated, and we found that many beneficiaries were able to work, go to school, or do housework (82.0%). However, 46 beneficiaries (14.6%) received home care, and 9 (2.9%) were living in hospitals or other medical facilities. During the last 6 months of the study, 10 beneficiaries (3.2%) were hospitalized and 266 beneficiaries (84.4%) made regular hospital visits. Overall, 240 beneficiaries (76.2%) had not obtained a physical disability certificate, whereas 59 (18.7%) received a Grade 1 certificate (most severe physical disability). Only 15 beneficiaries (4.8%) were reported as requiring care or support. At the time the forms were submitted, the clinical course of FD for 290 beneficiaries, as compared with the previous year, was reported as unchanged for 178 beneficiaries (61.4%), worse for 81 (27.9%), and improved or cured for 31 (10.7%). The distribution of beneficiary-related characteristics among men and women was similar, and no significant sex difference was observed.

**Table 3.** Characteristics of daily life, medical services, physical disability certification, care requirements, and clinical course

Beneficiary-related features		Men (*n* = 198)		Women (*n* = 117)		Total (*n* = 315)	
		
		Number	(%)	Number	(%)	Number	(%)
*Aspects of life*							
	Working, attending school, or performing housework	159	(82.4%)	92	(81.4%)	251	(82.0%)
	Home care	28	(14.5%)	18	(15.9%)	46	(14.6%)
	Hospitalization or admission	6	(3.1%)	3	(2.7%)	9	(2.9%)
	Not known	5	—	4	—	9	—
*Visit status*							
	Primarily inpatient	9	(4.7%)	1	(1.0%)	10	(3.4%)
	Half inpatient/half outpatient	12	(6.3%)	8	(7.6%)	20	(6.8%)
	Primarily outpatient	170	(89.0%)	96	(91.4%)	266	(89.9%)
	Other or not known	7	—	12	—	19	—
*Physical-disability certification and grade*							
	Not certified	148	(75.1%)	92	(78.6%)	240	(76.4%)
	Grade 1	42	(21.3%)	17	(14.5%)	59	(18.8%)
	Grade 2–5	7	(3.6%)	8	(6.8%)	15	(4.8%)
	Not known	1	—	0	—	1	—
*Care-requirement certification status*							
	Needing care or support	8	(4.1%)	7	(6.5%)	15	(5.0%)
	No need for care or support	185	(95.9%)	100	(93.5%)	285	(95.0%)
	Not known	5	—	10	—	15	—
*Self-rated clinical course as compared with previous year*							
	Worse	52	(28.3%)	29	(27.4%)	81	(27.9%)
	Unchanged	109	(59.2%)	69	(65.1%)	178	(61.4%)
	Improved or cured	23	(12.5%)	8	(7.5%)	31	(10.7%)
	Other or not known	14	—	11	—	25	—

No sex difference was observed.

## DISCUSSION

This is the first population-based study of FD in Japan, and we found that the prevalence of FD was 0.25 cases per 100 000. More men than women with FD were beneficiaries of the Japanese Specified Disease Treatment Research Program, prevalence among men was 1.78 times that among women, and mean age at registration and disease onset in men was lower than in women.

FD is an inherited glycolipid metabolic disorder in which glycolipids accumulate in many tissues because of a lack or decrease in the activity of α-galactosidase A. The gene locus for this enzyme is on the X chromosome (Xq21.33-q22), and inheritance is X-linked recessive,^13^ which explains the differences observed between men and women in the present study. Hemizygous males experience pain in the extremities, angiokeratoma, corneal clouding, and vascular disorders of the heart, kidneys, and brain, whereas presentation in heterozygous females varies from asymptomatic to severe disease. FD has a wide phenotypic spectrum with no clear genotype–phenotype correlation.^14^ Therefore, patients, particularly women, without characteristic symptoms or organ damage are sometimes misdiagnosed or diagnosed later, which likely contributed to the lower prevalence and later reporting of age at disease onset among women in the present study. This phenomenon has been widely reported in patients with FD.^6^

No large-scale or descriptive epidemiologic studies of FD have been conducted because it is very rare. In 1 study, FD was diagnosed in 21 of 432 male patients (4.9%) and 7 of 289 female patients (2.4%) in a population sample of 721 young and middle-aged patients (age, 18–55 years) with acute cerebral infarction.^15^ A second trial showed that in a population sample of 230 men (age, 55–72 years) receiving continuous treatment for left ventricular hypertrophy, FD was confirmed in 7 (3.0%).^16^ FD was also investigated in 696 patients (age, 19–95 years) undergoing dialysis for chronic kidney disease, among whom it was diagnosed in 4 of 401 (1.0%) male patients and 1 of 295 (0.3%) female patients.^17^ Similarly, a recent study of 1024 Japanese patients undergoing hemodialysis showed that 1 man and 2 women had FD.^8^ Programs to screen those at risk are likely to be effective in controlling FD by enabling the start of medical therapy before significant organ damage develops. This is vital in improving clinical outcomes and QOL, given the difficulties patients with FD face on a daily basis, as shown in our findings. A new method of identifying patients involves screening newborns. This was effective in a population of 37 104 Italian male infants: FD was diagnosed in 12 neonates (0.03%) in the 3 days after birth.^3^

To our knowledge, this is the first population-based study of the daily activities of the FD beneficiaries after introduction of enzyme replacement therapy. We found that more than 80% of both men and women were capable of attending work and school and performing tasks such as housework, despite sex differences in prevalence and age distribution. The small number of studies published before the widespread use of enzyme replacement therapy reported reduced QOL among FD patients, and the authors of those reports suggested that effective treatment with an appropriate agent would have great potential for improving QOL in patients with FD.^18^^,^^19^ Our data on the activities of FD beneficiaries appear to show improvement in the clinical course of FD patients after the start of therapy.

In the present study, the prevalence and standardized prevalence ratio were highest in the Tohoku region, followed by Koshinetsu/Hokuriku and Tokai. The prevalence of FD can be affected by factors such as the founder effect.^2^ A recent study assessed the influence of geographic location on FD phenotype among European women and showed that women living in northern countries tended to have higher severity scores than those living in southern countries.^20^ Interestingly, this was not seen in men, and the authors suggested that the findings were related to dietary and/or environmental influences. However, there are few studies of these factors, and although it is possible that environmental factors alter the course of FD and its overall prevalence, further investigation is clearly required to confirm this hypothesis.

The present analysis has some limitations. Although FD is a rare genetic disorder, and this study was a nationwide investigation (*n* = 315), the use of beneficiary data is likely to underestimate the total number of cases. Thus, the numbers may not accurately reflect overall prevalence. The present data indicate that the prevalence of FD in Japan was 0.25 per 100 000 individuals, which is much lower than figures published in other reports (0.85 and 2.5 per 100 000).^4^ It remains to be determined whether this difference is due to geographic differences and/or the use of different reporting systems.

In the present study, we calculated the prevalence of FD, ie, 0.25 cases per 100 000, and observed similar epidemiologic characteristics with regard to daily activity and clinical course among male and female FD beneficiaries, despite significant sex differences in prevalence and age distribution. Second, the high percentage (>80%) of individuals with FD who were attending work and school and performing tasks such as housework could reflect an improved clinical course for FD after enzyme replacement therapy. In addition, prevalence varied by region, although the explanation for this is unclear. We must continue data collection and conduct further investigations to improve our understanding of the descriptive epidemiology of FD.
